# Genetic rescue, the greater prairie chicken and the problem of conservation reliance in the Anthropocene

**DOI:** 10.1098/rsos.160736

**Published:** 2017-02-22

**Authors:** S. M. Mussmann, M. R. Douglas, W. J. B. Anthonysamy, M. A. Davis, S. A. Simpson, W. Louis, M. E. Douglas

**Affiliations:** 1 Biological Sciences, University of Arkansas, Fayetteville, AR, USA; 2 Illinois Natural History Survey, University of Illinois, Champaign, IL, USA; 3 Illinois Department of Natural Resources, Prairie Ridge State Natural Area, Newton, IL, USA; 4 Illinois Department of Natural Resources, Gibson City, IL, USA

**Keywords:** assignment test, genetic mark–recapture, management unit, microsatellite DNA, relatedness, *Tympanuchus cupido pinnatus*

## Abstract

A central question in conservation is how best to manage biodiversity, despite human domination of global processes (= Anthropocene). Common responses (i.e. translocations, genetic rescue) forestall potential extirpations, yet have an uncertain duration. A textbook example is the greater prairie chicken (GRPC: *Tympanuchus cupido pinnatus*), where translocations (1992–1998) seemingly rescued genetically depauperate Illinois populations. We re-evaluated this situation after two decades by genotyping 21 microsatellite loci from 1831 shed feathers across six leks in two counties over 4 years (2010–2013). Low migration rates (less than 1%) established each county as demographically independent, but with declining-population estimates (4 year average *N* = 79). Leks were genetically similar and significantly bottlenecked, with low effective population sizes (average *N*_e_ = 13.1; 4 year *N*_e_/*N* = 0.166). Genetic structure was defined by 12 significantly different family groups, with relatedness *r* = 0.31 > half-sib *r* = 0.25. Average heterozygosity, indicating short-term survival, did not differ among contemporary, pre- and post-translocated populations, whereas allelic diversity did. Our results, the natural history of GRPC (i.e. few leks, male dominance hierarchies) and its controlled immigration suggest demographic expansion rather than genetic rescue. Legal protection under the endangered species act (ESA) may enhance recovery, but could exacerbate political–economic concerns on how best to manage ‘conservation-reliant’ species, for which GRPC is now an exemplar.

## 1. Introduction

The Anthropocene (i.e. the human domination of natural global processes) is controversial in both definition and demarcation [1]. It has a serial record in North America, starting with the Beringian migration and continent-wide diversification, then European colonization, followed by westward expansion with its agricultural modifications and industrial enhancements, leading to the post-WW2 ‘great acceleration’ [2]. By most measures, it is best reflected in Midwestern North America, a region increasingly fragmented over the last 300+ years, with forests felled, prairies ploughed and streams sequestered for agricultural and urban purposes.

### 1.1. Impacts of the Anthropocene

Results are less controversial when impacts of the Anthropocene are measured on biodiversity. For example, many species are now listed as threatened and endangered (T&E) under the ESA (US Endangered Species Act), with recovery a prolonged process at best. Furthermore, 84% are now recognized as ‘conservation-reliant’ [3], meaning that direct and ongoing management will still be required even if recovery is achieved. This provokes an obvious query: ‘Given the surge in global threats, how best can species and their ecosystems be effectively managed?’ Mitigation strategies that blunt accumulating declines have indeed been proposed, with the foremost being assisted migration of species [4] and the intentional translocation of individuals [5]. The former aims to establish populations beyond their historic range, whereas the latter strives to initiate or augment populations within the native range.

### 1.2. Definitions and examples of genetic rescue and translocations

‘Genetic rescue’ [6] is a mitigation strategy whose intent is to restore genetic diversity and reduce extinction risks in small, isolated and frequently inbred populations. Its fundamental driver is translocation [7], a form of demographic rescue that adds numerically to a population so as to prevent its potential extinction. However, demographic and genetic rescue are often conflated, as each can increase population size and/or fitness [8]. This aspect, in turn, necessitates a more thorough definition, assessment and documentation of pre- and post-translocation genetic ancestry [9]. This is an important consideration in that declining ecological conditions, reductions in available habitat and the natural history of the species will conflate any evaluations of post-translocation genetic rescue [10].

Yet despite these caveats, genetic rescue is viewed as a positive conservation tool, largely owing to its perceived success with iconic species: greater prairie chicken [11], European adder [12], bighorn sheep [13] and Florida panther [14]. Each seemingly persisted as isolated, inbred populations with diminished reproductive success and declining demographics. Following translocation, their demographic rates were seemingly enhanced and recovery promoted.

### 1.3. Potential downsides and necessary re-evaluations

Yet translocations have drawbacks [15,16], as does the more directed genetic rescue, in that benefits may be temporary and with continuous monitoring required to assay for outbreeding depression or reduced effective population size. As an example, the genetic rescue of Isle Royale wolf coincided with a rapid reduction in food resources compounded by the insular nature of the environment, with the subsequent result being a population crash [17]. Of note is the occasional transitory nature of genetic rescue, as well as the necessity for guidelines that define its initiation and prolongation, particularly when supplementation is conducted in the face of deteriorating environmental conditions, an unfortunate occurrence for many populations of conservation concern [9,10]. These issues also surface in this study.

Interestingly, three of the four iconic species above revealed mixed results following re-examination. For European adder, the positive trajectory was reviewed [18] and subsequently posited as reflecting demographic rescue [9], whereas genetic rescue of bighorn sheep was confirmed [19], but with the effects of pre-translocation genetic drift readily apparent. Lastly, follow-up studies on Florida panther yielded conflicting and thus controversial results, but with ‘rescue’ seemingly promoted [20].

### 1.4. The current context

**Figure 1.**
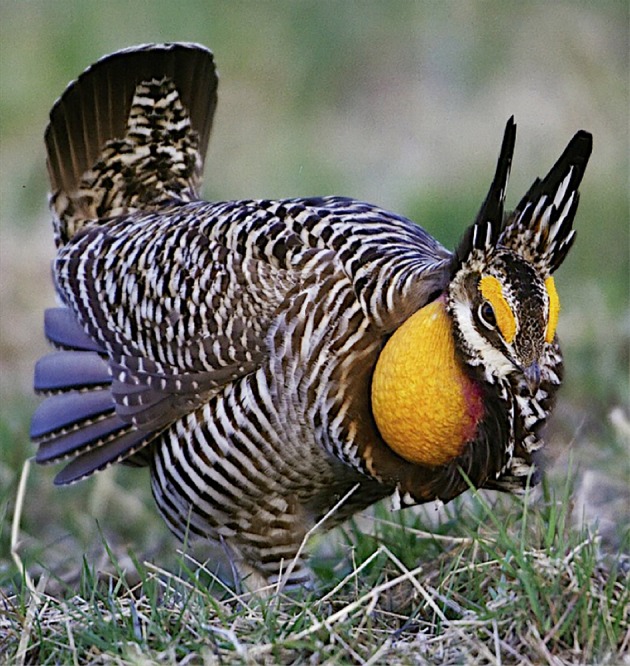
Male greater prairie chicken (*Tympanuchus cupido pinnatus*) engaged in a courtship display while on a booming ground in Jasper County, Illinois. Males expand their yellow-orange and scarlet-edged air sacs located on the sides of their necks so as to produce breeding calls. They also erect feathers on the nape and tail as part of a mating display. Photo is published with the permission of Richard Day, Daybreak Imagery (Alma IL, 62807; www.daybreakimagery.com/). It also appeared as the cover photo for *Illinois Audubon* (Fall, 2014; the Illinois Audubon Society, Springfield, IL, 62708).

Herein, we re-evaluated the fourth icon, the greater prairie chicken (GRPC: *Tympanuchus cupido pinnatus*; figure 1), a ground-nesting game bird whose natural history involves dominance polygyny (= lek-breeding, where males display in groups to attract a mate, with only a small subset subsequently reproducing). These aspects of its natural history provide ecological and evolutionary hurdles too difficult for genetic rescue to surmount [9]. GRPC was once widely distributed across the North American great plains but now persists in small remnants, thus necessitating intensive management [21]. In Illinois, GRPC declined precipitously from millions (mid-nineteenth century), to 2000 (1962), then 46 (1998) (electronic supplementary material, figure S1), provoking translocations from neighbouring states [9] that seemingly resuscitated its numbers (electronic supplementary material, part A). Consequently, it has been highlighted in journals [22] and textbooks [23–25] as an early and successful case of genetic rescue. However, annual declines were quickly apparent in the census number of males, with only 73 recorded in 2011 (electronic supplementary material, table S1). It is thus unclear if neutral genetic diversity was indeed successfully promoted. Much like the European adder, it again approaches an extinction vortex, with additional translocations initiated but subsequently suspended.

We sought to re-evaluate the population genetic status of GRPC within and among Illinois counties, so as to gauge the effectiveness of its genetic rescue and to statistically contrast its contemporary population genetic estimates with those pre- and immediately post-translocation. These analyses were unpublished, despite the recognition of GRPC as an icon of genetic rescue. In doing so, we tested the following hypotheses: (i) estimates of genetic variability for GRPC differ significantly between pre- and post-translocation; and (ii) post-translocation estimates do not differ significantly from those more contemporary. We then explore our findings within a broader context that involves the ESA and the issue of conservation-reliant species, and propose several potential mitigation strategies that may separate GRPC from its status as an exemplar of conservation reliance.

## 2. Material and methods

### 2.1. Samples, genotypes, gender and preliminary analyses

**Figure 2.**
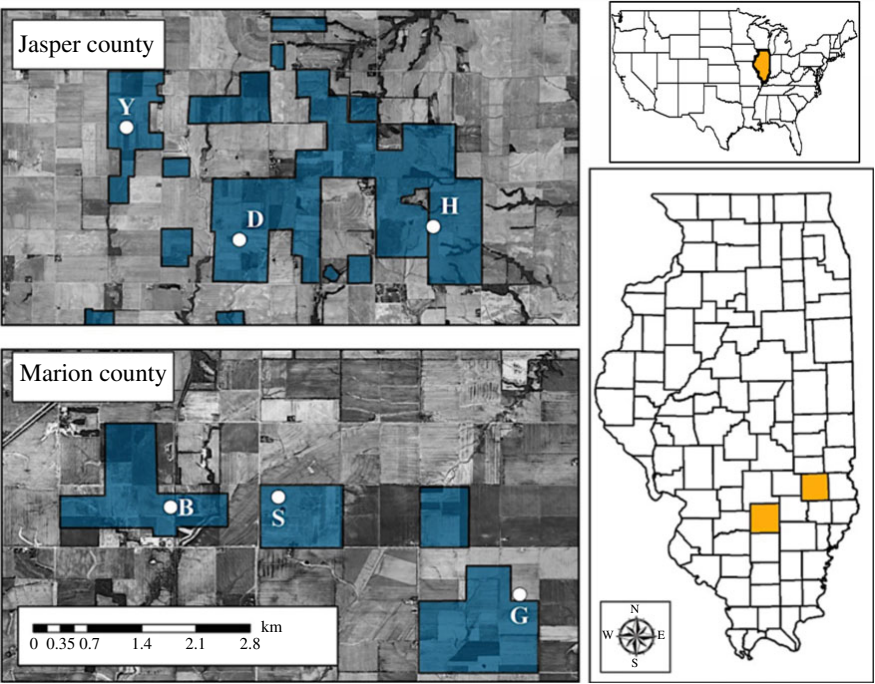
Maps display locations of leks for greater prairie chicken in two Illinois counties: Jasper and Marion (county locations designated in the Illinois map at bottom right, with Jasper County east and north of Marion County). Picture (above, right) depicts the location of Illinois in the United States. Feathers for genotyping were collected from six leks: Bainbridge (B), Guymon (G) and Survey (S) in Marion County (bottom left map), and Donsbach (D), Hunt (H) and YFM (Y) in Jasper County (top right map). Polygons shaded in blue represent lands managed by the Illinois Department of Natural Resources.

Approximately 4300 shed feathers were collected at six leks in Prairie Ridge State Natural Area (Marion and Jasper counties, Illinois; figure 2), with genomic DNA extracted from 3144 (73%) (table 1). We tested 83 microsatellite DNA loci originally developed for eight Galliform species, with 59 (71%) amplifying successfully in GRPC. Of these, 24 (41%) were combined into six multiplex panels of four loci each, with 21 yielding unambiguous genotypes. Our set of loci included the six originally employed in pre- and post-translocation analyses of Illinois GRPC [16]. Contemporary evaluations used both six- and 21-locus datasets, whereas pre- and post-comparisons used only the six-locus dataset.

**Table 1.** Yearly population size estimates for male greater prairie chicken (*Tympanuchus cupido pinnatus*) in Marion and Jasper counties, Illinois, as derived from genotypes generated across 21 microsatellite loci. Listed are year (of estimate); count = number of males observed on the leks; genotype = total number of distinct genotypes identified; CJS = Cormack–Jolly–Seber population estimate based on genotypes; s.e. = standard error for estimate. Statistical comparisons of count versus genotypes were non-significant.

year	count	feathers	genotypes	CJS	s.e.
2010	63	861	57	64.1	3.7
2011	46	865	37	38.3	1.4
2012	33	682	29	30.7	1.6
2013	27	736	22	23.7	1.7
Total	169	3144	123	156.8	8.4

Genotypes were condensed into unique individuals, and those samples with minor allelic differences were re-scored to identify and correct for potential genotyping errors. Additional details regarding extractions, amplifications, identification of genotypes and gender, tests for Hardy–Weinberg equilibrium (HWE) or linkage disequilibrium (LD), and rarefaction procedures for population genetic assays are provided in electronic supplementary material, part B.

### 2.2. Derivation of census, breeding and effective population sizes

Capture histories were imported into Rcapture [26], a package that applies the Cormack–Jolly–Seber mark–recapture model so as to estimate the census size (*N*_c_) of leks and counties. The mark/recapture of genotypes was then compared statistically with census estimates derived from visual counts of males at leks each year.

A linkage disequilibrium estimator (LdNe: [27]) was employed to gauge effective population size (*N*_e_) for each county and lek. Breeding population size (*N*_b_) was also estimated by county using a two-sex, no-sex change model in AgeNe [28], with the following parameters: a 5 year lifespan consisting of one non-reproductive and four reproductive years, a fecundity estimate of 0.32, brood size = 13, and annual survival for juveniles = 0.34 and 0.42 for adults [29]. Equal sex ratios were assumed and the Poisson factor was set at 2 to account for the high variance in reproductive success. From these estimates, we then derived *N*_b_/*N* and *N*_b_/*N*_e_ ratios for management relevance.

### 2.3. Population bottlenecks

Demographic parameters are important for long-term population persistence, yet are influenced by genetic attributes inherent to small populations [17]. Here we gauged recent population bottlenecks (i.e. less than five generations) by contrasting estimates of observed heterozygosity (*H*_o_) empirically derived from our microsatellite data against expected heterozygosity (*H*_e_) under Hardy–Weinberg mutation–drift equilibrium (HWE) [30]. A bottleneck was identified when *H*_o_ was significantly greater than *H*_e_ for a population under HWE. A variety of statistical approaches and models of microsatellite evolution were employed in our bottleneck analyses, the most powerful being the Wilcoxon signed-rank test [31] and the infinite alleles model [32]. A mode-shift test was also applied to evaluate the potential occurrence of a historic bottleneck within a few dozen generations (i.e. post-1990), with rejection manifested by an L-shaped frequency distribution of alleles.

### 2.4. Contemporary population structure, dispersal and relatedness

To assess genetic structure, an admixture model (Structure v. 2.3.3, [33]) was applied with no priors, correlated allele frequencies (CAFs) and *K* = 1–16 (where *K* = numbers of aggregates). To test for consistency, the program was run for 1 000 000 generations, with the first 100 000 as burn-in, and with 20 iterations per *K*-value. The appropriate *K*-value was determined by deriving Δ*K* [34] using Structure Harvester [35], and results then combined with Pr(*K*) so as to visualize population assignments [36]. Microsatellite data were also used to test for demographic independence among counties and leks [37].

Pairwise *F*_ST_ values were derived to assess genetic divergence among leks and counties, whereas isolation by distance (IBD) was tested using a Mantel test (GenAlex v. 6.5; [38]). Average relatedness (*r*) within and among leks was estimated with 1000 bootstrap replicates (CoAncestry; [39]). A contemporary likelihood approach was employed for the comparison of individual dyads against a third reference [40], with relatedness values [41] gauged as follows: unrelated (*r* = 0); second cousin (*r* = 0.063); first cousin (*r* = 0.125); half-sib (*r* = 0.25); parent/offspring or full-sib (*r* = 0.5). Average relatedness within and among gene pools was also derived and then tested for significance using a one-sample *t*-test [42]. The correlation between geographical distance and mean pairwise relatedness among leks was examined with a Mantel test (Genalex v. 6.5; [38]).

### 2.5. Temporal comparison of genetic diversity: 10 and 20 years post-translocation

All temporal comparisons employed the six-locus dataset, in that pre- and post-translocation values were derived solely from these loci [16]. Owing to a scarcity of samples, the pre-translocation estimate was calculated from 32 incidental mortalities of uncertain origin gathered over 19 years, whereas the post-translocation estimate was recorded only for Jasper County.

Mean values for observed heterozygosity (*H*_o_) and allelic richness (*A*_R_) were compared among pre-translocation, post-translocation and contemporary Marion and Jasper counties populations (2010–2013), using Welch's *t*-test for unequal variances. This approach is more robust than Student's *t*-test and maintains type I error rates despite unequal variances and sample sizes. Values for contemporary populations (2010–2013) were derived from our data, whereas estimates for pre- and post-translocation were calculated using original summary statistics [16] and a suitably modified web-based program (http://stats.stackexchange.com/questions/30394/how-to-perform-two-sample-t-tests-in-r-by-inputting-sample-statistics-rather-tha) executed in R [43]. The necessary standard deviations were calculated from standard errors: (http://www.ajdesigner.com/phpstatistics/standard_deviation_sample.php).

## 3. Results

### 3.1. Samples, genotypes and gender

After eliminating samples with missing data, the 3144 single-feather extractions yielded 1831 complete genotypes (58%). These represented 88 unique males (table 1), with 96% detected multiple times, 23 individuals (26%) detected over 2 years, 13 (15%) over 3 and 2 (2%) in all 4 years. Four individuals (5%) survived during 2010–2013.

### 3.2. Census, breeding and effective population sizes

Population estimates, as generated from the capture/recapture of male genotypes on each lek, did not differ significantly from those produced from visual count data (table 1). Estimates were doubled so as to obtain a yearly census number (*N*), under the assumption of a 1 : 1 gender ratio.

Estimates for *N*_e_ were uniformly low (overall average 13.1), with Jasper County *N*_e_ = 12.7 and Marion County *N*_e_ = 13.5 (electronic supplementary material, table S2). *N*_e_ for leks averaged 15.9 and varied between 2.9 and 38.4 (the latter based on only seven individuals). The number of breeding adults (*N*_b_) in Marion County was estimated as 24, whereas that for Jasper County was 17.

Ratios of *N*_e_/*N* effectively link demographic and evolutionary processes across a wide range of taxa, as reflected by the fact that half its variance is explained by only two life-history traits: age at maturity and adult lifespan [44]. Ratios of *N*_b_/*N*, *N*_e_/*N* and *N*_b_/*N*_e_, as averaged over both counties, were quite low. The 4 year average for GRPC (i.e. *N*_e_/*N* = 0.189) is considerably lower than that recorded for sage grouse (*N*_e_/*N* = 0.574; table S2 in [44]).

### 3.3. Population structure, dispersal, relatedness and bottlenecks

Remarkable lek fidelity was apparent among recaptures, with 89% (i.e. 34/38) resident each year at the same lek. *F*_ST_ estimates were significantly different between counties, but not among leks within counties (table 2). Linear distances among leks were significantly correlated with *F*_ST_ values, validating the existence of IBD.

**Table 2.** Pairwise *F*_ST_ estimates calculated between leks of greater prairie chicken located in Jasper and Marion counties, Illinois. Estimates were derived using the full microsatellite DNA set of 21 loci (above diagonal) and the reduced set of six loci (below diagonal). Leks are assigned to counties by placing (J) or (M) following the name, where J = Jasper County and M = Marion County. Statistical significance is displayed with an asterisk. Leks are significantly different between counties (i.e. (J) versus (M)) but not within counties (i.e. (J) versus (J) or (M) versus (M)).

leks	Donsbach (J)	Hunt (J)	YFM (J)	Bainbridge (M)	Guymon (M)	Survey (M)
Donsbach (J)	X	0.007	0.004	0.108*	0.110*	0.149*
Hunt (J)	0.005	X	0.00	0.094*	0.096*	0.141*
YFM (J)	0.009	0.022	X	0.083*	0.094*	0.125*
Bainbridge (M)	0.072*	0.126*	0.086*	X	0.009	0.04
Guymon (M)	0.082*	0.112*	0.108*	0.011	X	0.035
Survey (M)	0.100*	0.165*	0.109*	0.033	0.036	X

The mean migration rate averaged 0.9% between Marion and Jasper counties, establishing each as a demographically independent unit [45] (electronic supplementary material, figure S2, top). Migration rates among leks within counties were higher, averaging 11.2%.

**Figure 3.**
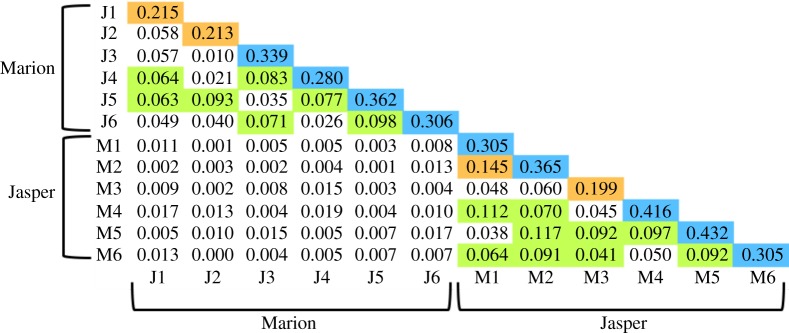
Pairwise relatedness estimates calculated among family groups of greater prairie chicken in Illinois. Calculations were based on 21 microsatellite loci. J1–J6 = family groups in Jasper County; M1–M6 = family groups in Marion County. Colours highlight relatedness values that exceed minimum values for the following relationships: blue = half-sib (*r* > 0.25); orange = first cousin (*r* > 0.125); green = second cousin (*r* > 0.0625).

Each population was subdivided into six gene pools that equated to family groups (*N* = 12; electronic supplementary material, figure S2, bottom) rather than leks. Family groups were unrelated between counties, yet differed significantly within each across both 21- or six-locus datasets (electronic supplementary material, table S3). Average relatedness within and between counties exceeded that of half-sib (i.e. *r* = 0.25), with Jasper *r* = 0.286 and Marion *r* = 0.337. The average for both counties was *r* = 0.311 (figure 3). Patterns of relatedness among leks were statistically consistent with linear distance (Mantel test, *p* < 0.006). Even though individuals reflected high lek fidelity, family groups did not, with most evenly distributed among leks within respective counties, a pattern consistent with natal dispersal.

Both counties showed statistically significant evidence for recent and historic bottlenecks. Four leks were significant for recent bottlenecks, whereas the remaining two contained too few individuals for a valid test. Three (of four) leks also reflected the signatures of a historic bottleneck (electronic supplementary material, table S2).

### 3.4. Comparison of genetic diversity at 10 and 20 years post-translocation

The 21- and six-locus datasets (electronic supplementary material, table S4) were used in the calculation of contemporary (2010–2013) genetic diversity by lek and county, and pre-/post-translocation diversity by county (Jasper 2003; table 3*a*). Heterozygosity is a reliable gauge for the loss of genetic variation and a good predictor of the potential for a population to evolve. However, our pairwise comparisons did not differ significantly among pre-, post- and contemporary evaluations (table 3*b*). Pre-translocation *A*_R_ for Jasper County differed significantly from post-translocation and contemporary estimates (2010–2013; table 3*c*). However, statistical significance can be questioned, given the undefined locations of the pre-translocation samples and the broad disparity in the timing of collection.

**Table 3.** (*a*) Mean values for observed heterozygosity (*H*_O_), expected heterozygosity (*H*_E_) and allelic richness (*A*_R_), with standard errors and standard deviations, as derived from six microsatellite DNA loci amplified in greater prairie chicken in Illinois. IL-Pre = pre-translocation; IL-Post = post-translocation; M/10–13 = Marion County (2010–2013); J/10–13 = Jasper County (2010–2013). (*b*) Results of Welch's unequal variances *t*-test comparing values of *H*_O_ among samples. Bottom triangle = *t*-values, whereas top triangle = probability values. (*c*) Results of Welch's unequal variances t-test comparing values of *A*_R_ (allelic richness) among samples, with information as in (*b*). Significant *t*-values (and probabilities) at Bonferroni-adjusted alpha = 0.008 are highlighted in italics. *N* = sample size; *H*_O_ = observed heterozygosity; *H*_O_s.e. = standard error for observed heterozygosity; *H*_O_s.d. = standard deviation for observed heterozygosity; *H*_E_ = expected heterozygosity; *H*_E_s.e. = standard error for expected heterozygosity; *H*_E_s.d. = standard deviation for expected heterozygosity; *A*_R_ = allelic richness; *A*_R_s.e. = standard error for allelic richness; *A*_R_s.d. = standard deviation for allelic richness.

	*N*	*H*_O_	*H*_O_s.e.	*H*_O_s.d.	*H*_E_	*H*_E_s.e.	*H*_E_s.d.	A_R_	A_R_s.e.	A_R_s.d.
(*a*) sample										
IL-Pre	32	0.525	0.09	0.9266	0.654	0.055	0.5222	4.7	0.9	1.0832
IL-Post	18	0.611	0.08	0.5347	0.676	0.058	0.3744	5.5	0.6	0.4628
M/10–13	40	0.58	0.13	1.374	0.547	0.113	1.3065	5.1	0.79	0.9797
J/10–13	48	0.734	0.08	0.774	0.688	0.068	0.6848	5.6	0.83	1.0269

	IL-Pre	IL-Post	M/10–13	J/10–13						
(*b*) *H*o										
IL-Pre	X	>0.68	>0.79	>0.21						
IL-Post	0.42	X	>0.92	>0.68						
M/10–13	0.27	0.1	X	>0.35						
J/10–13	1.27	0.42	0.93	X						
(*c*) *A*_R_										
IL-Pre	X	*<0.0007*	>0.11	*<0.0004*						
IL-Post	*3.63*	X	<0.04	>0.59						
M/10–13	1.62	2.11	X	<0.022						
J/10–13	*3.72*	0.54	2.33	X						

## 4. Discussion

Illinois GRPC is recognized as a conservation icon and a textbook example of ‘genetic rescue’ in that translocations seemingly promoted an increase in fitness as gauged by a population expansion post-translocation [11]. In addition, follow-up studies [16] identified diminishing prairie habitat as a factor that may limit recovery. Our study, conducted some 20+ years post-translocation, calculated population demographic and genetic parameters over multiple time frames, then quantified and statistically tested ‘rescue’ effects in Illinois GRPC for the first time. Our analyses showed that pre- and post-translocation results deviated but little. The post-translocation response was similar in magnitude to other (statistically non-significant) rescue attempts, one of which included GRPC in Wisconsin (table 1 of [46]). This led us to instead propose demographic expansion in lieu of genetic rescue.

In our study, individual genotypes were verified using gender-specific markers. We then contrasted our genetically derived mark/recapture estimates against visual counts of individuals on leks, and by so doing detected male longevity extending over multiple years. Our estimates of the breeding population (*N*_b_) and effective population (*N*_e_) sizes allowed us to derive contemporary management parameters such as ratios of *N*_b_/*N*, *N*_e_/*N* and *N*_b_/*N*_e_. We also established the demographic independence of populations by quantifying immigration rates, and our estimates of genetic relatedness clearly underscored the management liabilities inherent to the mating system of GRPC, with only a few dominant males successfully reproducing.

More importantly, we quantified those population genetic parameters for GRPC that have been missing to date, such as pairwise *F*_ST_-values, and identified the magnitude and extent of historic (i.e. post-1990) and contemporary bottlenecks (i.e. less than five generations). Our results suggest that genetic rescue may have been an optimistic interpretation for GRPC, with genetic drift and a demographic expansion via translocation implicated as overriding factors [9,10]. In hindsight, translocation and demographic expansion yielded results for GRPC that were unsustainable in the near term, with conservation reliance promoted instead as a management issue.

Below, we elaborate on our findings in more detail, particularly with regard to those extrinsic and intrinsic factors that integrate with the life history and ecology of GRPC and which promote its conservation reliance. We then offer several potential solutions that may contravene its status and promote recovery in Illinois.

### 4.1. Defining the matrix of conservation reliance

**Figure 4.**
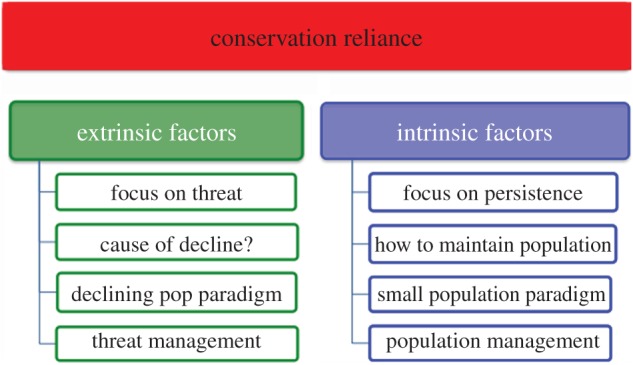
Conceptual depiction of conservation reliance within the context of causation and remediation. Causation can be influenced by both intrinsic and extrinsic factors, with a focus on either persistence or threat, and with remediation efforts being informed by small versus declining-population paradigms.

A key objective when managing conservation-reliant species such as GRPC is to restore its fitness environment to a state that existed before its decline. Here, two options prevail: ‘threat management’ (i.e. extrinsic to the species) and ‘population management’ (i.e. intrinsic to it) [3] (figure 4). Threat management works under the assumption that appropriate ecological remediation can eventually reverse declines, whereas population management focuses on life-history attributes of a species, recognizing that recovery can be impeded by the inflexibility of certain characteristics such as a conserved niche. Extrinsic and intrinsic factors often act in synergy and, by so doing, incorporate the ‘declining-’ and ‘small-population’ paradigms first introduced by Caughley [47] (per figure 4).

Extrinsic factors are inherent to the declining-population paradigm, where numerical reductions are induced by habitat fragmentation, introduced predators, over-harvest or related impacts. Subsequent questions might focus on: How was the decline initiated? Is it perpetuated? Can it be rectified? In contrast, intrinsic factors are inherent to the small-population paradigm, with emphases on inbreeding, low effective population size and demographic isolation (among others). It is theory-rich (e.g. extinction-vortex; [48]) and recognizes the mutual reinforcement of biotic and abiotic drivers. Although genetic and/or modelling endeavours are promoted as a means of remediation, their practicality is often weakly associated [49]. Importantly, insights can be gleaned with regard to causation and remediation when both paradigms are effectively juxtaposed (figure 4).

### 4.2. Conservation reliance, extrinsic factors and the ecological theatre

Many of the management issues that underlie the conservation reliance of GRPC pertain to extrinsic factors (i.e. ‘threats’). Given this, a ‘threat management’ approach would address low abundances, with rehabilitation and/or extension of critical habitat as potential mitigation.

GRPC is an ecological specialist with relatively narrow and inflexible habitat preferences [50] that underscore niche conservatism [51]. It lacks the plasticity to adjust to the alterations of the North American tall grass prairie, and is consequently confined to isolated prairie remnants within an expansive agricultural matrix (figure 2). Only two populations remain in Illinois, and our analyses identified each as being significantly different and demographically independent (i.e. ‘management units’; [45]).

Colonization would be facilitated and gene flow promoted if dispersal occurred among existing habitat fragments, but this process is severely constrained by the ecological characteristics of grouse. For example, more than 50% of yearling greater sage grouse will disperse to a non-natal lek (a positive characteristic), but with a downside that they remain faithful to it in subsequent years [52]. Our molecular mark–recapture analyses confirmed this pattern in GRPC, with most individuals not only resident over the study period at a single lek, but also with few movements between leks. Such limited dispersal promotes the development of localized kin clusters, a second characteristic of grouse that was subsequently identified in our analyses. GRPC in Illinois persists as significantly different family groups, yet are dispersed within counties such that leks are rendered genetically similar (figure 3). This negates the benefits of natal dispersal [53], and instead promotes inbreeding.

**Figure 5.**
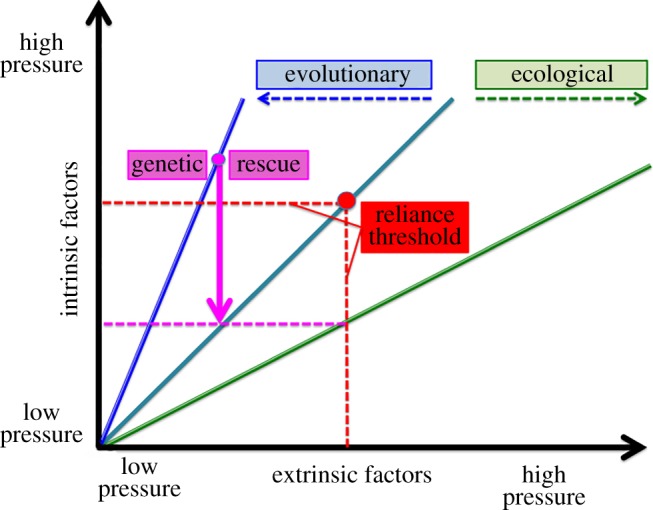
The persistence and decline of populations are influenced by factors both extrinsic (horizontal axis) and intrinsic (vertical axis), with extinction risk increasing as pressure intensifies from lesser to greater. The situation where intrinsic and extrinsic pressures contribute equally to the risk of extinction is depicted by the 45° line (teal-coloured). However, the life history of a species (blue line) or its ecological attributes (green line) may constrain remediation and thus increase the risk of extinction, as represented by deflection towards the appropriate intrinsic/extrinsic axis. Genetic rescue (pink arrow) is a remediation effort that can potentially diminish extinction risk to a value below the threshold for conservation reliance (red-dotted line). While this is accomplished by ameliorating small-population effects, it does not substantially reduce ecological threats.

Habitat reductions and loss of connectivity are extrinsic factors reflected in the declining-population paradigm, yet their impacts can be modulated by intrinsic factors such as ecological specialization and dispersal behaviour. This, in turn, underscores the synergy among intrinsic and extrinsic factors (figure 4), and how their juxtaposition begets conservation reliance (figure 5).

### 4.3. Conservation reliance, intrinsic factors and the evolutionary play

The intrinsic factors of conservation reliance are often reflected in the natural history of a species. They impede its capacity to persist within an anthropogenic-dominated landscape, and point to the small-population paradigm as a driver of conservation reliance. In this context, we interpret previous remediation strategies for GRPC as largely unsuccessful in that they worked against those limitations imposed by the life history of the species. This aspect has been overshadowed in GRPC by the benefits accrued from demographic expansion [9,10].

As an example, lekking is a reproductive strategy that sustains the lifetime reproductive success of a few dominant males, as demonstrated by the fact that copulations are achieved by less than 20% of resident GRPC males [54,55]. In addition, lek fidelity in male lesser prairie chicken (*T. pallidicinctus*) increases with age and thus reinforces consanguinity, but with declining survivorship [56]. Interestingly, our analyses detected considerable variance in male longevity, with several genetically identified males surviving over 4 years. Our analyses also demonstrated the negative impacts of male dominance and longevity on effective population (*N*_e_) size, yet with few management solutions short of artificial propagation.

Female fitness in lek-mating species is often at parity with that of males, and for several reasons: the existence of female dominance hierarchies [54]; a tendency with maturation for females to mate consanguineously [56]; an elevated number of nest failures [57] and a high predation rate on chicks, with less than 20% surviving until day 21 [58]. However, female lesser prairie chicken often have a much lower survival rate than males, largely owing to nest predation [59], a characteristic that can diminish female reproductive output.

Other gender-based variances in life-history promote subsidiary issues: females maintain larger home ranges, disperse farther and more frequently, and are thus more susceptible to the vagaries of climate change and a fragmented habitat [54,60]. They also maintain activity centres within 5 km of leks [61], suggesting that effective conservation efforts should focus not only on active leks but also on nearby habitat. These aspects have obvious population genetic ramifications and thus relate to the small-population paradigm, but they also reinforce the interplay among extrinsic and intrinsic factors (figure 4), and how this has impeded recovery. In this sense, a successful remediation would involve aspects that fall at opposite ends of the conservation–reliance continuum, thus reflecting the complexity of the issue.

### 4.4. Genetic rescue, translocation and conservation reliance

The relevance of extrinsic and intrinsic factors is mediated by the ecology and evolutionary history of a species (figure 5). Here, genetic rescue addresses population persistence in an attempt to depress conservation reliance below a ‘reliance’ threshold, thus reducing the pressures exerted by intrinsic factors. Yet, there is also scant capacity (if any) to remedy extrinsic factors, thus limiting the effect. Two factors establish the baseline for conservation reliance, and both must be alleviated in tandem (figure 5).

Was genetic rescue a success for GRPC in Illinois? Major caveats prevent a definitive answer. Pre-translocation genetic estimates were derived from 32 incidental mortalities without county of origin gathered over 19 years (i.e. approx. 0.6 sample per year or approx. one sample every 2 years). Unfortunately, these data were the only option available for the derivation of comparative metrics [16], and indeed, this is acknowledged. However, numbers of GRPC fluctuated considerably during the 19 year period within which sampling occurred (electronic supplementary material, table S1). A bottleneck over a relatively short span of time during this period would eliminate rare alleles and reduce allelic richness, but not seriously impact heterozygosity [62]. As the pre-translocation population became gradually smaller over time, genetic diversity was lost through drift. All of the above renders as equivocal the baseline against which post-translocation effects were gauged, and argues that criteria supporting genetic rescue be re-evaluated, particularly in the context of a limited habitat (defined here as leks) and a population expansion promoted by translocation [10]. This also necessitates the documentation of genetic change following rescue [9].

As a management approach, translocation seemingly addresses both extrinsic and intrinsic factors. It underpins genetic rescue, has roots in population genetic theory (i.e. ‘small-population paradigm’), invokes immigration as a mechanism for ecological remediation (i.e. ‘declining population’) and has been promoted as effective in recovering GRPC in Illinois [16]. However, serious limitations are revealed under closer examination. For example, numerous ‘census migrants’ [63] are needed so as to ensure the potential of at least one genetically effective migrant to counterbalance the aforementioned variance in individual reproductive success. This is particularly difficult when game birds are involved. Translocated individuals depart quickly and remain largely unaccounted for unless radio-tracked during extensive post-translocation meanders [64,65]. In addition, survival for translocated individuals is much lower, even when acclimation is properly done [66], a phenomenon largely attributed to stress [67].

GRPC translocations in Illinois have a history of setbacks (electronic supplementary material, part A), with success either ambiguous or transitory, neither of which is sustainable. Translocations may avoid the costs of restoring connectivity but also invoke long-term intensive management (i.e. the perpetuation of conservation reliance). This will in turn, tax recovery such that it becomes unsustainable from an economic and political stance [68]. In this sense, GRPC is a conservation icon not because of an ambiguous genetic rescue, but rather, as an exemplar of a conservation-reliant species.

### 4.5. Can short-term solutions facilitate long-term success?

Self-sustaining populations of GRPC in Illinois may be an elusive endpoint, particularly given the severe constraints imposed by ecological and evolutionary characteristics. A similar situation exists for the lesser prairie chicken [69]. A reduction in its habitat is uncorrectable in the near term, yet most certainly exerts a strong influence on the reproductive success of dominant, lekking individuals [70].

Are potential solutions indeed plausible for GRPC? We consider four. Listing under the ESA affords legal protection for those populations that face extinction in parts of their range. In Illinois, GRPC is ‘state-endangered’ but federal listing would leverage increased ecosystem management, much as with the congeneric lesser prairie chicken. The ESA also offers protection for ‘distinct population segments’, and both populations qualify as they are demographically independent and significantly different from one another. An elevated listing would also reaffirm the ‘state acres for wildlife enhancement’ programme implemented by the Illinois Department of Natural Resources in an attempt to convert agricultural land back into prairie (http://www.dnr.state.il.us/orc/safe/). This, in turn, would promote critical habitat for GRPC, as well as long-term connectivity among prairie remnants. However, one particular downside for ESA protection is that its regulatory restrictions may be perceived negatively by private landowners, thus impeding appropriate management.

Second, GRPC requires large tracts of intact native grassland and, as such, would represent an ‘umbrella species’ for the conservation of the North American prairie (where umbrella species is defined as one whose extensive habitat requirements encompass many other biodiversity components, and whose protection would yield a generalized effect). Other unique endemics within this ecosystem should also be promoted for legal protection. This, in turn, would underscore the distinctiveness of the ecosystem, promote its public awareness and shape stakeholder perceptions regarding its conservation.

Third, GRPC is particularly vulnerable to climate change, as typified by its steep decline during the Anthropocene. Species so recognized demonstrate seven life-history characteristics [71], five of which (71%) are manifest in GRPC: it occurs in restricted habitats, has specific habitat requirements, low reproductive rates, limited dispersal capabilities and low genetic variability. Empirical evidence for climate-related impacts is provided by the lesser prairie chicken, whose abundances track climatic events that are short term and extreme rather than long term, and with numbers promoted by wet springs but retarded by warmer, drier summers [72].

Midwestern North America now supports an elevated and energy-intensive economy whose greenhouse gas emissions exceed the national average by more than 20% [71]. Furthermore, regional crop production will accelerate over the next few decades in response to elevated CO_2_ levels and an extended growing season, but to the detriment of prairie landscapes [73]. Clearly, global climate change will be an additional challenge to the persistence of GRPC, and its impacts on the ecosystem would qualify as a component of risk analysis under the ESA [74].

Fourth, entrepreneurial response would emphasize that translocations occur from within rather than external to Illinois. This could avoid the political and economic difficulties involved in multistate translocations. In this sense, an important management action would be to restore connectivity among the two Illinois populations, so as to mitigate the genetic effects that stem concomitantly from prolonged bottlenecks and gradual population losses over an extended period. A bilateral introduction of individuals from one population to another would be a mechanism that can foster connectivity. However, populations are precariously small and significantly different as currently diagnosed. Given these caveats, an *ex situ* propagation strategy may be a potential solution (e.g. whooping crane, *Grus americana*; [75]).

## 5. Conclusion

Conservation threats in the Anthropocene are most often a result of extrinsic factors that are potentially manageable, but within a cost/benefit framework encompassing triage as an endpoint (where triage is viewed as a conservation strategy that provides the greatest benefit for multiple species and ecosystems). Additional complications occur when habitat has been subsumed by agriculture, and where protected areas are insubstantial in their number or dimensions to sustain a population expansion (as herein). However, restoring the vast North American prairie to a historic benchmark (i.e. revising an external threat) is clearly unachievable in that the prairie has already been replaced by an agro-urban patchwork. In a similar fashion, those approaches that solely address intrinsic factors (i.e. the small-population paradigm) also have a low probability of success and, as a consequence, promote rather than alleviate conservation reliance.

However, it is counterproductive to question the success or failure of translocations and genetic rescue (i.e. small-population approaches). Each is admirable with regard to effort [16], and instructive with regard to the depth of analyses elicited (this study). Yet, successful baselines are defined by their endpoints, and indeed if management must extend into the future. State-endangered species (as herein) are just as conservation-reliant as are those federally listed [76], with little concern for priority in that threats often exceed the probability of being ameliorated by the legal process [68]. The only difference is the opportunity for potential management of state-listed species that can preclude the federal listing process [77].

A major challenge for mitigation of GRPC, is the development of stakeholder initiatives that will drive its management at ecologically relevant scales. In this sense, we now have a clear understanding of GRPC, its population genetic status, and those extrinsic and intrinsic factors that tether it to conservation reliance. We must now assemble the economic coalition that will match the needs of management with the contemporary constraints of the private sector, and by so doing develop a conservation plan that will alleviate its conservation reliance.

## Supplementary Material

Supporting Information for Mussmann et al.

## Data accessibility

Our data are deposited at the Dryad Digital Repository: http://dx.doi.org/10.5061/dryad.dr71n [78].
