# Supplementary material for: Genetic rescue, the greater prairie chicken and the problem of conservation reliance in the Anthropocene
Source: R Soc Open Sci. 2017 Feb 22;4(2):160736. doi: 10.1098/rsos.160736 (PMC5367285; doi:10.1098/rsos.160736)
Supplement: Supporting Information for Mussmann et al. [file rsos160736supp1.pdf]

The datasets supporting this article have been uploaded as part of the Supplementary Material

---

Supporting Information for Mussmann et al.:
"Genetic Rescue, the Greater Prairie Chicken, and the Problem of Conservation-Reliance in the Anthropocene"

Parts of this SI section:

A. Thirty six-year history of Greater Prairie Chicken translocations in Illinois
B. Details of methodology and results
C. References for supporting information
  - Figures S1-S2
  - Tables S1-S4

A. Thirty six-year history of Greater Prairie Chicken translocations in Illinois

The first translocation was made in 1979, when 57 Greater Prairie Chicken (GRPC) from Kansas were released in Illinois (Sparling 1979). Ultimately, 35 of these birds were relocated onto reclaimed strip-mine land in Perry County, IL, and nine were fitted with radio transmitters. Despite this, none were relocated five weeks post-release. Nine mortalities (26%) were known to have occurred, and no lek was established. In 1991, a successful translocation was conducted within Illinois by swapping two egg clutches between Marion and Jasper counties (Westemeier et al. 1991). Twelve translocated eggs hatched in Jasper County, while eight were successful in Marion County. Movement over the intervening 60km between the two sites is possible, but not commonplace (Westemeier & Jansen 1995).

Following failed attempts to save the Illinois population through habitat protection and intensive management of exotic species, birds were again translocated from Kansas, Minnesota,

and Nebraska (Westemeier et al. 1998). Between 1992 and 1998, 506 GRPC were released into Jasper and Marion counties in seven yearly translocations [(S.A. Simpson – Personal Communication) (figure S1, table S1)] and these were deemed successful in rescuing the populations from the brink of extinction. Censuses indicated 25-67% of transplanted birds integrated into the breeding population. Hatching success rose from 76% to 94% post-translocation, and mean fertility rates increased from 91% to 99% (Westemeier et al. 1998).

The loss of neutral genetic diversity in Illinois GRPC was documented through a comparison of pre-bottleneck museum specimens with larger, contemporary populations (Bouzat 2000). Initial studies evaluated individuals across six microsatellite loci originally developed for Chicken (*Gallus gallus*). Post-bottleneck populations in Illinois had fewer alleles per locus (3.7) and reduced heterozygosity (0.57) when contrasted with populations in Kansas (5.8/ 0.60), Minnesota (5.3/ 0.65), and Nebraska (5.8/ 0.63) (Bouzat et al. 1998a). Genetic analysis of Illinois museum specimens discovered several alleles found in both historical specimens and extant populations but which were lacking in the post-bottleneck population in Illinois (Bouzat et al. 1998b). Furthermore, the historical specimens also possessed alleles that were absent in extant populations.

Subsequent application of these same six microsatellite loci for assessment of populations in Wisconsin prompted the discovery of genotyping problems with three loci (Bellinger et al. 2003). These were consequently replaced with three new loci developed for Red Grouse (*Lagopus lagopus scoticus*). This updated suite of loci was later applied to pre- and post-bottleneck Illinois specimens, as well as to samples collected in 2003 (Bouzat et al. 2009). Results using the new set of loci suggested that translocations successfully increased allelic richness (A_R) (5.5) and expected heterozygosity (H_E) (0.68) in the 2003 population relative to

that found in pre-translocation samples (4.7/ 0.65) (table 2). However, these values remained low relative to larger populations such as those in Kansas (8.4/ 0.76) and Nebraska (8.0/ 0.73) (Bouzat et al. 2009).

B. Details of the methodology and results

Sample collection, DNA extraction and amplification

The QIAamp DNA Micro Kit (Qiagen-CAT# 56304) was used for DNA extraction (protocol DY04 Aug-06), under sterile conditions with dedicated equipment inside a dead air box (AirClean Systems 600 PCR Workstation). Microsatellite PCR conditions were: 10µl polymerase chain reaction (PCR) volumes containing 2µl Go-*taq* 5x buffer (PROMEGA), 2.0mM MgCl2, 0.25mM dNTPs, 0.2µg BSA, 1 unit Go-*taq* DNA polymerase (PROMEGA), 0.1uM each of forward and reverse primer, and approximately 20ng template DNA. Annealing temperatures for PCR varied by locus, while cycling conditions were identical and consisted of: Initial denaturation step at 94°C for 3min, followed by 15 cycles of 94°C for 45s, annealing for 45s, 72°C for 30s, followed by 25 cycles of 94°C for 30s, annealing for 30s, and 72°C for 45s, with a final extension of 72°C for 3min. All DNA concentrations were diluted to 5ng/µl. Amplification was confirmed using 2.5% agarose gels stained with a 3X solution of GelGreen nucleic acid stain (BIOTIUM).

Genotypes, gender, and preliminary analyses

Samples were first screened using a subset of six loci to test for amplification success, and resulting complete genotypes (N=1,831, 58%) were condensed into unique individuals using GENECAP (Wilberg & Dreher 2004) and ALLELEMATCH (Galpern et al. 2012). Both programs

were used to detect scoring errors by identifying genotypes that differed by 1-2 allele differences. GENECAP was utilized to generate capture histories of all unique genotypes; each 'capture' was verified by genotyping the additional 15 loci (i.e., across all 21 loci). Probability of two individuals (P_{ID}) sharing the same genotype (Paetkau & Strobeck 1994) was calculated using GENALEX V6.5 (Peakall & Smouse 2012).

To determine gender of unique genotypes, multiple sets of primers were tested (Griffiths et al. 1998, Kahn et al. 1998, Fridolfsson & Ellegren 1999, Bantock et al. 2008) to amplify sex-linked loci. One successful primer set (P8/M5; Griffiths et al. 1998, Bantock et al. 2008) was used. PCR conditions were: 10µl volumes containing 2µl Go-*taq* 5x buffer (PROMEGA), 2.0mM $MgCl_2$, 0.25mM dNTPs, 0.2µg BSA, 1 unit Go-*taq* DNA polymerase (PROMEGA), 10.0mM each of forward and reverse primer, and approximately 10ng template DNA. The thermal profile was: initial denaturation at 94°C for 3min, followed by 10 cycles of 94°C for 45s, annealing at 54°C for 45s, and a 72°C elongation for 45s, followed by an additional 30 cycles of 94°C for 30s, annealing at 54°C for 30s, and a 72°C elongation for 30s, followed by a final extension at 72°C for 5min.

MICROCHECKER v2.2.3 (Van Oosterhout et al. 2004) was utilized to detect null alleles and scoring errors while GENEPOP v4.1 (Raymond & Rousset 1995, Rousset 2008) was employed to test for deviations from Hardy-Weinberg equilibrium (HWE) and linkage disequilibrium (LD) with significance gauged using a Bonferroni adjusted P-value. Rarefaction (HP-RARE: Kalinowski 2004) was applied to compensate for variation in sample size when calculating allelic richness (A_R). Lek estimates were calculated for seven individuals (the smallest lek sample size), while county A_R estimates were made for 18 individuals to facilitate comparisons with values from previous studies. Pairwise F_{ST} values were calculated to assess

genetic differences among leks and counties, while isolation by distance (IBD) was tested using a Mantel test in GENALEX v6.5.

Results for genotypes, gender, and mark-recapture

Extracting and amplifying DNA from feathers was successful but inconsistent among years (31%-71%), and comparable to other studies (i.e., 56% and 69%). Across all years, 88 unique genotypes were identified, with 96% detected multiple times. The PID was low (Jasper = 2.5×10^{-20}, Marion = 5.4×10^{-18}) indicating that unique individuals did not share the same genotype. All individuals visiting a lek were not sampled, but results across years do reflect observed demographic trends. For example, 63 males were observed across all leks in 2010, with 56 identified from shed feathers. Fewer individuals were detected per year as the study progressed, and this was consistent with genotyping results (fewer birds = fewer feathers).

Sex-identification was reliable, in that a single gender-specific fragment amplifies in males, whereas two in females. These data were verified using known individuals from Kansas. In Illinois, all unique samples amplified but a single fragment and were thus identified as male. Given this, population estimates derived from capture histories could then be directly compared to count estimates from lek surveys.

Jolly-Seber mark-recapture estimates were similar to male-based observation surveys at leks. Estimates for 2010 approximated the observed number of male GRPC (64.1 vs. 63), whereas those in 2011-2013 were slightly less than lek counts (table 2).

C. References for supporting information

Bantock TM, Prys-Jones RP, Lee PLM. 2008 New and improved molecular sexing methods for museum bird specimens. *Mol. Ecol. Resour.* **8**, 519—528.

Bellinger MR, Johnson JA, Toepfer J, Dunn P. 2003 Loss of genetic variation in greater prairie chickens following a population bottleneck in Wisconsin, USA. *Conserv. Biol.* **17**, 717—724.

Bouzat JL. 2000 The importance of control populations for the identification and management of genetic diversity. *Genetica* **110**, 109—115.

Bouzat JL, Cheng HH, Lewin HA, Westemeier RL, Brawn JD, Paige KN. 1998a Genetic evaluation of a demographic bottleneck in the greater prairie chicken. *Conserv. Biol.* **12**, 836—843.

Bouzat JL, Lewin HA, Paige KN. 1998b The ghost of genetic diversity past: Historical DNA analysis of the greater prairie chicken. *Am. Nat.* **152**, 1—6.

Bouzat JL, Johnson JA, Toepfer JE, Simpson SA, Esker TL, Westemeier RL. 2009 Beyond the beneficial effects of translocations as an effective tool for the genetic restoration of isolated populations. *Conserv. Genet.* **10**, 191—201. (doi:10.1007/s10592-008-9547-8).

Fridolfsson K, Ellegren H. 1999 A simple and universal method for molecular sexing of non-ratite birds. *J. Avian Biol.* **30**, 116—121.

Galpern P, Manseau M, Hettinga P, Smith K, Wilson P. 2012 ALLELEMATCH: an R package for identifying unique multilocus genotypes where genotyping error and missing data may be present. *Mol. Ecol. Resour.* **12**, 771—778.

Griffiths R, Double MC, Orr K, Dawson RJG. 1998 A DNA test to sex most birds. *Mol. Ecol.* **7**, 1071—1075.

Kahn NW, St. John J, Quinn TW. 1998 Chromosome-specific intron size differences in the avian CHD gene provide an efficient method for sex identification in birds. *The Auk* **115**, 1074—1078.

Kalinowski ST. 2004 Counting alleles with rarefaction: private alleles and hierarchical sampling designs. *Conserv. Genet.* **5**, 539—543.

Paetkau D, Strobeck C. 1994 Microsatellite analysis of genetic variation in black bear populations. *Mol. Ecol.* **3**, 489—495.

Peakall R, Smouse PE. 2012 GENALEX 6.5: Genetic analysis in Excel. Population genetic software for teaching and research-an update. *Bioinformatics* **28**, 2537—2539. (doi:10.1093/bioinformatics/bts460).

Raymond M, Rousset F. 1995 GENEPOP (version 1.2) - population genetics software for exact tests and ecumenicism. *J. Hered.* **86**, 248—249.

Rousset F. 2008 GENEPOP ' 007: a complete re-implementation of the GENEPOP software for Windows and Linux. *Mol. Ecol. Resour.* **8**, 103—106.

Sparling DW (1979) Restoration of greater prairie chickens in southwestern Illinois: project summary October 1978 - July 1979. Southern Illinois University Cooperative Wildlife Research Laboratory, Carbondale, IL.

Van Oosterhout C, Hutchinson WF, Wills DPM, Shipley P. 2004 MICROCHECKER: software for identifying and correcting genotyping errors in microsatellite data. *Mol. Ecol. Notes* **4**, 535—538.

Westemeier RL, Jansen RW. 1995 Non-resident prairie chickens in Illinois. *IL Nat. Hist. Surv. Rept*. **332**, 1—9.

Westemeier RL, Simpson SA, Cooper DA. 1991 Successful exchange of prairie chicken eggs between nests in two remnant populations. *Wilson Bull*. **103**, 717—720.

Westemeier RL, Brawn JD, Simpson SA, Esker TL, Jansen RW, Walk JW, Kershner EL, Bouzat JL, Paige KN. 1998 Tracking the long-term decline and recovery of an isolated population. *Science* **282**, 1695—1698.

Wilberg MJ, Dreher BP. 2004 GENECAP: a program for analysis of multilocus genotype data for non-invasive sampling and capture-recapture population estimation. *Mol. Ecol. Notes* **4**, 783—785.

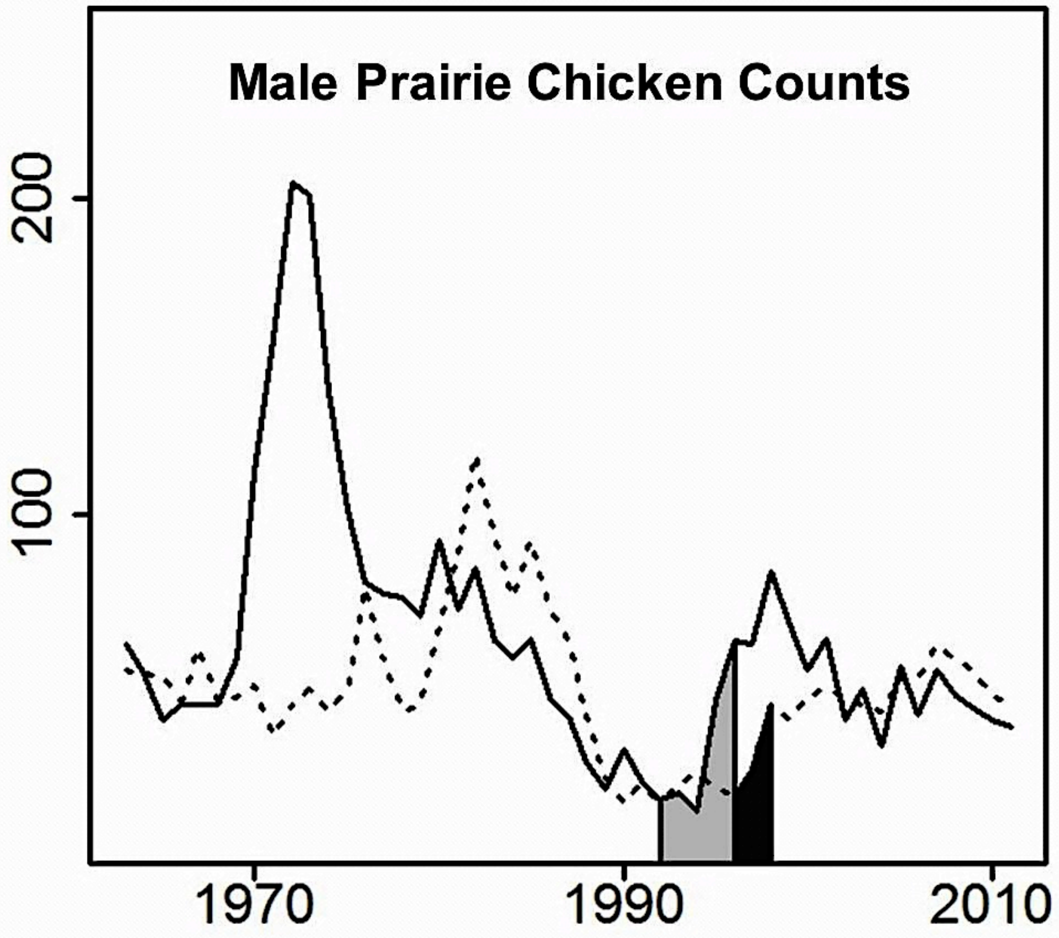

Figure S1. Numbers of male Greater Prairie Chicken observed in each year in Marion (broken line) and Jasper (solid line) counties, Illinois. Lek surveys have been conducted annually since 1963 (X axis), with only males being counted (Y axis). Translocation programs occurred from 1992-1996 (Jasper – shaded grey) and 1996-1998 (Marion – shaded black). Data provided by S.A. Simpson, Illinois Department of Natural Resources.

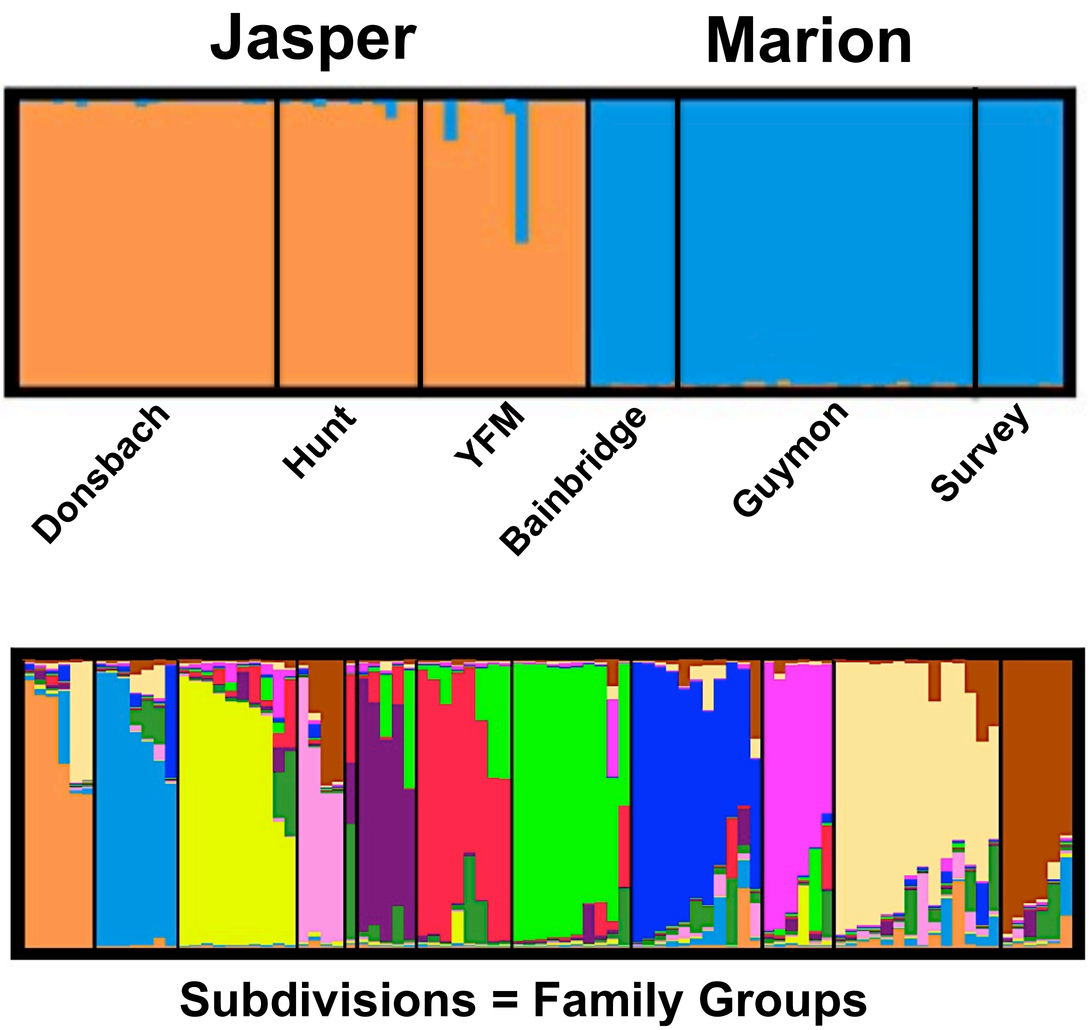

Figure S2. Probabilities of assigning individual Greater Prairie Chicken to group (*K*) as determined by posterior probability [Pr(*K*)]. The correlated allele frequencies (=CAF) model was applied in STRUCTURE 2.3.4 using 21 microsatellite DNA loci. Colors represent distinct gene pools and each vertical bar corresponds to an individual. The best definition of population structure (top) and family structure (bottom) was selected according to ΔK. Top: K=2 clusters correspond to Jasper (orange) and Marion (blue) counties, with X-axis designating lek of origin. Bottom: K=12 clusters correspond to family groups with average relatedness r=0.311, exceeding minimum value for half-sibs (r=0.25).

Table S1. Translocations of Greater Prairie Chicken into Illinois from 1992 to 1998. The number of birds listed each year is by county (A = Jasper; B = Marion). Male/ Female = gender of released birds; Kansas, Minnesota, Nebraska = source.

**A. Jasper**						
	**Kansas**		**Minnesota**		**Nebraska**	
Year	Male	Female	Male	Female	Male	Female
1992	-	-	-	15	-	-
1993	-	-	8	4	-	-
1994	46	50	-	-	-	-
1995	48	50	-	-	-	-
1996	-	-	-	-	25	25
**B. Marion**						
1996	-	-	-	-	24	24
1997	45	47	-	-	-	-
1998	46	49	-	-	-	-

Table S2. Probability values for results of bottleneck tests, and estimates for effective population size (N_e) derived by County (A) and Lek (B). All bottleneck tests are based on the infinite alleles model (IAM) of microsatellite DNA evolution. Sign Test = All loci fit IAM mutation-drift equilibrium; SDT = Standardized Difference Test, with same hypothesis as Sign Test; Wilcoxon = Wilcoxon Rank Test, with same hypothesis as Sign Test; Mode-Shift = Test for historic bottleneck, where 'L-Shape' = normal and 'Shifted' = bottleneck. Non-significant probability values are in bold; Leks with asterisk contain too few of individuals to appropriately conduct bottleneck test; 95% CI = Confidence interval.

	County/Lek	Sign Test	SDT	Wilcoxon	Mode-Shift	N_e (95% CI)
**A.**	Jasper	0	0	0	L-Shape	13.5 (12.1/15.1)
	Marion	0.0039	0.0017	0.0047	L-Shape	12.7 (11.1/14.5)
**B.**	Donsbach	0.0001	0	0	L-Shape	10.5 (8.7/12.8)
	Hunt	**0.0532**	0.0069	0.0045	Shifted	22.5 (14.0/46.7)
	YFM	0.001	0	0	Shifted	10.9 (8.4/14.4)
	Bainbridge*	**0.3976**	**0.206**	**0.1439**	L-Shape	38.4 (12.5/∞)
	Guymon	0.0491	0.0055	0.0021	L-Shape	10.3 (8.6/12.3)
	Survey*	0.0106	0.0025	0.0012	Shifted	2.9 (1.9/7.9)

Table S3. Mean relatedness (r) within six leks of Greater Prairie Chicken in Illinois as derived from (A) the full set of microsatellite loci (21-locus set) and (B) the reduced set (6-locus set), following methods of Wang (2007). All relatedness values are significantly different from zero within leks (one-sample t-test). Relatedness >half-sib=***; > 1st cousin=**; > 2nd cousin=*.

A. Lek (21)	r	Variance	t	d. f.	P
Donsbach	0.108*	0.019	12.36	252	0.0001
Hunt	0.082*	0.014	4.61	44	0.0001
YFM	0.079*	0.015	6.55	104	0.0001
Bainbridge	0.107*	0.018	3.58	20	0.0019
Guymon	0.108*	0.021	13.05	299	0.0001
Survey	0.313***	0.043	6.76	20	0.0001
B. Lek (6)					
Donsbach	0.095*	0.022	10.25	252	0.0001
YFM	0.109*	0.031	6.34	104	0.0001
Hunt	0.132**	0.027	5.32	44	0.0001
Bainbridge	0.176**	0.027	4.83	20	0.0001
Guymon	0.186**	0.043	15.52	299	0.0001
Survey	0.316***	0.027	8.56	20	0.0001

21-locus set: r=0.797/ av. =0.133

06-locus set: r=1.014/ av. =0.169

Table S4. Genetic diversity estimates for Greater Prairie Chickens found within leks and counties in Illinois. (A), (C) were derived using the full 21-locus set, as designated by the number following the header, whereas (B), (D) were derived from a reduced 6-locus set, also so designated. Estimates in (D) for pre-translocation and 'Jasper 2003' are from Bouzat et al. (2009). N = sample size; A_M = mean number of alleles per locus; A_R = allelic richness; A_{pr} = private alleles; H_O = observed heterozygosity; H_OSE = standard error for H_O; H_E = expected heterozygosity; H_ESE = standard error associated with H_E; "-" = data unavailable.

**(A)** Lek (21)	N	A_M	A_R	A_{pr}	H_O	H_OSE	H_E	H_ESE
Donsbach	23	5.7	4.4	3	0.71	0.04	0.67	0.04
Hunt	10	5.4	4.9	5	0.72	0.05	0.66	0.04
YFM	15	5.6	4.7	2	0.75	0.05	0.69	0.03
Bainbridge	7	4.4	4.4	2	0.63	0.05	0.60	0.04
Guymon	25	5.8	4.3	4	0.64	0.06	0.60	0.05
Survey	7	3.2	3.2	1	0.63	0.08	0.50	0.06
**(B)** Lek (6)								
Donsbach	23	5.8	4.6	0	0.70	0.07	0.66	0.08
Hunt	10	5.6	4.9	2	0.70	0.13	0.63	0.08
YFM	15	5.8	4.9	0	0.81	0.07	0.71	0.05
Bainbridge	7	4	4	1	0.63	0.10	0.54	0.08
Guymon	25	4.4	3.5	1	0.55	0.13	0.54	0.12
Survey	7	3	3	0	0.57	0.17	0.48	0.13
**(C)** County (21)								
Marion 2010-13	40	6.2	4.6	38	0.64	0.06	0.61	0.05
Jasper 2010-13	48	6.5	4.9	45	0.72	0.04	0.69	0.04
**(D)** County (6)								
Marion 2010-13	40	5	3.9	4	0.58	0.13	0.55	0.11
Jasper 2010-13	48	6.6	5.1	12	0.73	0.08	0.69	0.07
Jasper 2003	18	5.5	5.5	-	-	-	0.68	0.06
Pre-Translocation	32	5.2	4.7	-	-	-	0.65	0.06
